# Retrospective Analysis of eFAST Exams to Detect COVID‐19 in Trauma Patients

**DOI:** 10.1155/emmi/6173255

**Published:** 2026-07-10

**Authors:** Nicholas Gossett, Michelle Thao Nguyen, Roy Almog, Andy Hsueh, Soheil Saadat, Megan Guy, Edmund Hsu, Madison Nashu, Ronald Goubert, Amanda Dos Santos, Andy Nguyen, Matthew Whited, Jessa Baker, John Christian Fox

**Affiliations:** ^1^ School of Medicine, University of California Irvine, Orange, California, USA, uci.edu; ^2^ Department of Emergency Medicine, University of California Irvine, Orange, California, USA, uci.edu; ^3^ Samaritan Healthcare, Moses Lake, Washington, USA; ^4^ Temecula Valley Hospital, Temecula, California, USA; ^5^ Eisenhower Medical Center, Rancho Mirage, California, USA; ^6^ Tibor Rubin VA Medical Center, Long Beach, California, USA

**Keywords:** COVID-19, eFAST, POCUS, RT-PCR, trauma

## Abstract

**Background:**

This original research study evaluates the utility of pulmonary point‐of‐care ultrasound (POCUS) within the extended focused assessment with sonography in trauma (eFAST) exam to detect asymptomatic COVID‐19 in emergency department trauma patients. Specifically, it examines whether lung findings, such as B‐lines, pleural thickening, and subpleural consolidations, can indicate a COVID‐19 infection.

**Methods:**

This retrospective review includes trauma patients aged 18 years or older who underwent eFAST and COVID‐19 swab testing at the University of California, Irvine Medical Center, from December 2020 to October 2022. Two blinded reviewers analyzed eFAST scans for more than two B‐lines, irregular pleural interface, or subpleural consolidations, with discrepancies resolved by ultrasound fellows. Sensitivity, specificity, and predictive values were calculated with 95% confidence intervals (CIs).

**Results:**

A total of 152 patients were included. Of 41 eFAST scans positive for COVID‐19 findings, six were confirmed by PCR testing, yielding a positive predictive value of 15.6% (CI: 7.9%–25.6%). Among the 111 eFAST scans negative for COVID‐19, 10 were PCR‐positive, giving a negative predictive value of 91.0% (CI: 84.1%–95.6%). Sensitivity was 37.5% (CI: 15.2%–64.6%), and specificity was 74.3% (CI: 66.1%–81.4%).

**Conclusion:**

Lung POCUS within the eFAST exam is not a reliable tool for detecting asymptomatic COVID‐19 infection in trauma patients due to limited sensitivity and low positive predictive value. Standard diagnostic methods, such as PCR testing, and the use of personal protective equipment should remain the primary approach to protect healthcare providers.

## 1. Introduction

As sporadic outbreaks of COVID‐19 recur, screening for COVID‐19 in the emergency department remains crucial for rapidly identifying individuals at risk of severe illness and preventing the spread of infection among patients and healthcare workers [1]. Although real‐time polymerase chain reaction (RT‐PCR) remains the standard for COVID‐19 screening, the availability of PCR test kits has decreased since the peak of the pandemic, with access becoming more limited to clinical settings [2]. Additionally, while PCR tests remain highly accurate against new variants, factors such as sampling technique, viral load, and test sensitivity can affect their performance and sensitivity, prompting further investigation into alternative methods [3]. This retrospective original research study aims to assess whether pulmonary point‐of‐care ultrasound (POCUS) within the extended focused assessment with sonography in trauma (eFAST) exam can identify asymptomatic COVID‐19 patients, providing a potential means of protecting providers from exposure and allowing healthcare workers to implement early safety measures.

Trauma resuscitation protocols commonly incorporate eFAST exams, involving the acquisition of subxiphoid, right upper quadrant, left upper quadrant, suprapubic, and thoracic ultrasonography windows to evaluate for intra‐abdominal and intra‐thoracic injuries. Ultrasound is a nonionizing, portable, and rapid diagnostic tool with no risks to patients. Although the eFAST exam was designed to rapidly assess unstable trauma patients, it has broader applications in detecting acute pulmonary pathology by concurrently capturing images of the lungs [4].

The eFAST exam traditionally evaluates trauma patients for intra‐abdominal free fluid and thoracic injuries, including pneumothorax, hemothorax, and pericardial effusion through a series of ultrasound views. Primarily, its use in detecting lung pathology has been limited to assessing traumatic injury. However, visualization of the lung fields during the lung component of the eFAST exam can reveal features associated with other pulmonary conditions, such as pneumonia or pulmonary edema. While not intended for comprehensive lung evaluation, eFAST could still detect pathologic lung findings such as B‐lines, pleural irregularities, and subpleural considerations. These features have been seen in viral pneumonia, including COVID‐19. Prior studies have demonstrated the potential use of lung ultrasound in identifying these findings, even in patients with asymptomatic or mild COVID‐19 infections [5, 6].

Recent studies highlight lung POCUS as a promising tool for early COVID‐19 detection and evaluation in the emergency department, offering advantages such as optimizing resource use, triaging based on the severity of lung findings, monitoring therapeutic effects, facilitating ventilation weaning, and minimizing healthcare professionals’ COVID‐19 exposure [5]. Furthermore, previous studies have utilized ultrasound to identify nonspecific lung findings such as B‐lines, consolidations, pleural thickening, and pleural effusions in patients hospitalized with COVID‐19 [6]. While ultrasound is sensitive in detecting lung pathology, interpreting eFAST may be challenging in patients with COVID‐19 pneumonia. It is often uncertain whether the observed B‐lines are incidental, stemming from underlying viral pneumonia, or related to pulmonary contusions, which may also cause pulmonary edema in the context of blunt or penetrating trauma [7]. Therefore, the reliability of POCUS lung findings from an eFAST exam in detecting COVID‐19 for trauma patients remains uncertain.

This study aims to determine if lung POCUS within the eFAST exam can reliably detect COVID‐19 in asymptomatic patients with moderate to critical trauma, potentially providing a means of rapid detection and thereby protecting healthcare providers from exposure.

## 2. Methods

### 2.1. Study Setting and Populations

This retrospective study reviewed electronic medical records of patients treated in the Emergency Department at the University of California, Irvine Medical Center in Orange, California, between December 2020 and October 2022. This is an academic, urban, Level 1 trauma center with an annual volume of approximately 65,000 patients. The inclusion criteria were participants aged 18 or older who underwent an eFAST exam during trauma activation and received COVID‐19 swab testing. This study was reviewed and approved by the Institutional Review Board of the University of California, Irvine (HS#: 2021‐6433).

For the purposes of this study, asymptomatic patients were defined as those with no documented respiratory symptoms and fever at initial trauma evaluation in the emergency department. However, given that this was a retrospective study, we could not definitively distinguish between truly asymptomatic presentations or assess symptom evolution after the initial ED presentation.

### 2.2. Study Protocol

This study was conducted and reported in accordance with the Standards for Reporting Diagnostic Accuracy Studies (STARD) 2015 guidelines. Two blinded, independent reviewers analyzed the lung portion of the eFAST scan and were blinded to the COVID‐19 test results. A positive study was defined as one containing > 2 B‐lines, irregular visceral‐to‐parietal pleural interface, and/or subpleural consolidations, all of which can be indicative of COVID‐19 infection. These diagnostic criteria were chosen based on previous studies that described common sonographic features of COVID‐19 pneumonia. Multiple B‐lines, irregular pleural lines, and subpleural consolidations have been repeatedly observed in patients with COVID‐19. These criteria align with findings reported by previous studies, which describe these ultrasound patterns as suggestive indicators of COVID‐19–related lung involvement [5–8]. Ultrasound scans that lacked any of these findings were considered to have screened negative for COVID‐19. In the five cases where there were disagreements between the reviewers regarding the presence of COVID‐19 findings in the lung ultrasounds, resolution was achieved through consultation with emergency medicine clinical ultrasound fellows.

The lung portion of the eFAST exam was primarily obtained using the linear probe and was performed by trained emergency medicine residents, fellows, and attending physicians. In most cases, operators obtained anterior lung views over 1‐2 rib spaces, with scan durations typically lasting between 2 and 5 s per side. The number of lung zones obtained varied depending on the operator but usually included only limited anterior thoracic views. The variability in operator expertise and imaging technique likely contributed to inconsistencies in diagnostic accuracy.

The timing between the eFAST scan and RT‐PCR testing was not standardized. Both assessments were performed during the same emergency department encounter as part of routine trauma evaluation workflow. All patients received an eFAST exam within minutes of arrival, while RT‐PCR testing was obtained when patients were clinically stable and according to staff availability. Exact times of testing were not collected for data analysis.

### 2.3. Data Analysis

Sensitivity, specificity, positive predictive values, and negative predictive values are presented as percentages, along with 95% confidence intervals (CIs). STATA 14 (StataCorp. 2015. Statistical Software: Release 14. College Station, TX: StataCorp LP) was used for data analysis, and the “diagti” command was utilized to calculate test specifications and CIs.

## 3. Results

Sensitivity, specificity, predictive values, and likelihood ratios are summarized in Table 1.

**TABLE 1:** *N* = 152 (with eFAST scan and COVID‐19 test results).

	Point estimate	95% confidence interval	
Sensitivity	37.50%	15.20%	64.60%
Specificity	74.30%	66.10%	81.40%
ROC area (Sens. + Spec.)/2	0.559	0.431	0.687
Likelihood ratio (+)	1.46	0.728	2.92
Likelihood ratio (−)	0.842	0.569	1.25
Positive predictive value	14.60%	5.57%	29.20%
Negative predictive value	91%	84.10%	95.60%

A retrospective analysis was conducted on 285 trauma patient charts from December 2020 to October 2022. Among these, 133 charts lacked either an associated eFAST scan or COVID‐19 PCR result, resulting in 152 enrollments with both eFAST and COVID‐19 results. Of these 152 enrollments, 41 were found to have lung eFAST scans that were positive for signs of COVID‐19.

**FIGURE 1:**
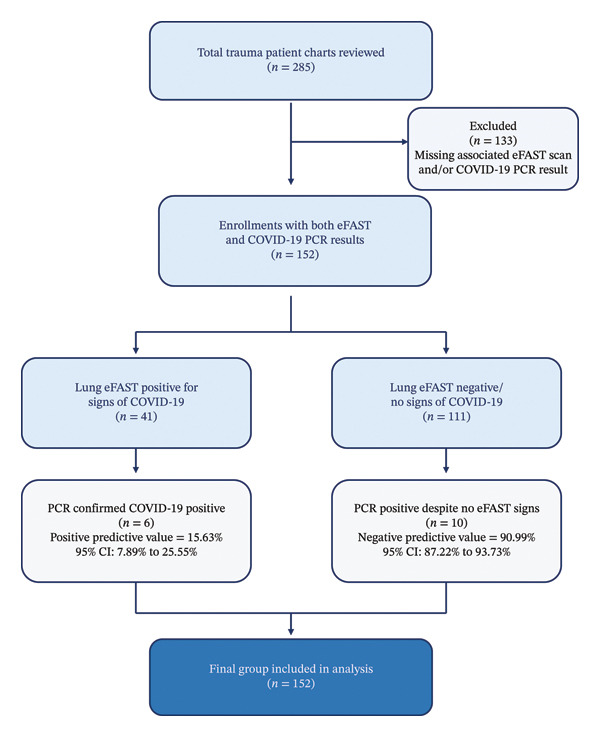
Study enrollment flow diagram.

Of the 41 eFAST scans with positive signs for COVID‐19, six were confirmed positive by COVID‐19 PCR testing, yielding a positive predictive value of 15.63% (CI: 7.89%–25.55%). Conversely, among the 111 eFAST scans with no signs of COVID‐19, 10 had a positive COVID‐19 PCR result, yielding a negative predictive value of 90.99% (CI: 87.22%–93.73%). The study enrollment process and patient selection are shown in Figure 1.

The sensitivity and specificity of the lung eFAST scan for COVID‐19 infection were found to be 37.50% (CI: 15.20%–64.57%) and 74.26% (CI: 66.07%–81.37%), respectively. The positive likelihood ratio (LR+) was 1.46 (CI: 0.73–2.92), indicating that a positive eFAST result is 1.46 times more likely to occur in patients with COVID‐19 than those without. The negative likelihood ratio (LR−) was 0.84 (CI: 0.57–1.25), suggesting a negative eFAST result is 0.84 times less likely to occur in patients with COVID‐19 than those without.

## 4. Discussion

After the COVID‐19 pandemic, strategies to diagnose the infection rapidly and effectively have become paramount. This study investigated the use of readily available POCUS as a testing modality to diagnose incidental COVID‐19 during the pandemic in patients presenting as a trauma activation. In our analysis, we evaluated COVID‐19 infection by identifying sonographic features—such as multiple B‐lines, pleural irregularities, and subpleural consolidations—that have been previously established as findings of COVID‐19 in prior studies [4–8]. By examining the correlation between COVID‐19 lung findings on eFAST and RT‐PCR results, this study aimed to establish the reliability of the eFAST exam in predicting COVID‐19 cases. Additionally, it explored the frequency of COVID‐19 in non‐COVID‐19–related trauma cases, offering broader implications for eFAST use in a variety of patient scenarios. These implications include but are not limited to early identification of COVID‐19, facilitation of timely patient isolation, expansion of emergency screening capabilities, and optimization of resource allocation, particularly in settings with limited access to dedicated COVID‐19 testing [1–3, 9, 10]. A key focus of this study was on asymptomatic trauma patients who were incidentally found to be COVID‐19–positive through PCR testing. This focus on asymptomatic patients reflects the real‐world challenge of identifying infection in patients who may not present with overt symptoms but still pose a risk of transmission.

The study assessed the specificity and sensitivity of eFAST in detecting COVID‐19–related features, exploring its potential as an early screening tool. A positive ultrasound result from our study was associated with a relatively low probability of actual COVID‐19 infection. The high negative predictive value was attributable to the point prevalence of COVID‐19 in our sample, which is 10.53% (CI: 6.14%–16.53%). Additionally, the eFAST scan shows limited effectiveness in predicting COVID‐19. With a LR+ of 1.46, a positive eFAST result slightly increases the probability of COVID‐19, but not enough to confirm the diagnosis confidently. Similarly, the LR− of 0.84 means that a negative result only slightly reduces the likelihood of the disease, making it ineffective for reliably ruling out COVID‐19. Based on our study’s results, ultrasound appears to have limited reliability for accurately identifying COVID‐19.

A key challenge was the variability in scan quality due to multiple operators, which complicated interpretation; for example, some lung scans lasted only a couple of seconds and generally focused on a single area of the lung, raising questions about whether this was sufficient for accurate assessment. Additionally, the lack of implementation and understanding of standardized protocols to identify COVID‐19 signs in POCUS remains a limitation.

Furthermore, selection bias may have been present because RT‐PCR testing was not performed for all patients, as eFAST scans were originally obtained from trauma cases without specific respiratory or COVID‐19 indications. This selection may have underrepresented COVID‐19–positive cases, potentially affecting the apparent accuracy of ultrasound findings for COVID‐19 detection. Verification bias may have further affected the results because PCR testing was not performed systematically across all patients. As a result, patients who received confirmatory RT‐PCR testing may not have been fully representative of the broader trauma population. This could have influenced the apparent generalizability across patients with varied clinical presentations. These limitations suggest that while ultrasound offers benefits in specific contexts, it should not be relied upon as a standalone diagnostic tool for COVID‐19. The findings underscore the need for continued reliance on established diagnostic methods, such as PCR testing, and the further development of guidelines for integrating POCUS into COVID‐19 diagnostic workflows.

Another limitation is the inability to classify COVID‐19 positive patients by disease stage, such as asymptomatic, presymptomatic, or symptomatic. Because symptom assessment relied on evaluation at the time of ED presentation, it is possible that some patients were in the incubation period or developed symptoms after imaging [11–13]. This limits our findings and may lead to an underestimation of ultrasound sensitivity in more advanced disease.

Finally, trauma‐related pulmonary conditions such as pulmonary contusions, hemothorax, atelectasis, and aspiration pneumonitis may have introduced confounding effects because they can produce ultrasound features that resemble those seen in viral pneumonia, including B‐lines, pleural irregularities, and subpleural consolidations [14–16]. This is consistent with prior reports cautioning that lung ultrasound findings described in COVID‐19 are not specific and may overlap with other interstitial or inflammatory lung processes [16]. Our study did not distinguish between trauma‐related and infection‐related ultrasound findings. This overlap may have made image interpretation more difficult and likely contributed to the low specificity and limited positive predictive value.

Future research should evaluate whether ultrasound has a meaningful role as an adjunctive screening tool in emergency department populations using standardized lung ultrasound protocols, consistent multizone imaging, defined sonographic criteria, and systematic PCR testing for all patients. Larger prospective studies should also account for operator variability, scan quality, and trauma‐related pulmonary conditions such as contusions, hemothorax, atelectasis, and aspiration pneumonitis, which may confound interpretation by producing findings like viral pneumonia. Until stronger evidence is available, ultrasound should remain an adjunct rather than a replacement for PPE, standard precautions, or established diagnostic testing in the ED.

In summary, our findings did not support the diagnostic value of POCUS within the eFAST exam for detecting asymptomatic COVID‐19 in trauma patients. Therefore, it should not be relied on to protect healthcare providers.

## Funding

The authors have nothing to report.

## Conflicts of Interest

The authors declare no conflicts of interest.

## Data Availability

The data that support the findings of this study are available from the corresponding author upon reasonable request.
